# ANGPTL1 attenuates colorectal cancer metastasis by up-regulating microRNA-138

**DOI:** 10.1186/s13046-017-0548-7

**Published:** 2017-06-12

**Authors:** Haiyan Chen, Qian Xiao, Yeting Hu, Liubo Chen, Kai Jiang, Yang Tang, Yinuo Tan, Wangxiong Hu, Zhanhuai Wang, Jinjie He, Yue Liu, Yibo Cai, Qi Yang, Kefeng Ding

**Affiliations:** 1 Department of Surgical Oncology, The Second Affiliated Hospital of Zhejiang University School of Medicine, 88 Jiefang Road, Hangzhou, Zhejiang 310009 China; 2 The Key Laboratory of Cancer Prevention and Intervention of China National Ministry of Education, The Key Laboratory of Molecular Biology in Medical Sciences of Zhejiang Province, Cancer Institute, Hangzhou, Zhejiang China

**Keywords:** Colorectal cancer, ANGPTL1, Metastasis, MicroRNA-138, Mechanism

## Abstract

**Background:**

Angiopoietin-like protein 1 (ANGPTL1) has been reported to suppress migration and invasion in lung and breast cancer, acting as a novel tumor suppressor candidate. Nevertheless, its effects on colorectal cancer (CRC) remain poorly defined. In this study, we aim to demonstrate the biological function of ANGPTL1 in CRC cells.

**Methods:**

We explored ANGPTL1 mRNA expression in human CRC tissues and its association with prognosis. CRC cell lines overexpressing ANGPTL1 or with ANGPTL1 knocked down were constructed and analyzed for changes in proliferation, colony formation, migration and invasion. ANGPTL1-regulated microRNAs were analyzed, and microRNA inhibitor and mimics were used to explore the role of microRNA in ANGPTL1-associated biological function.

**Results:**

ANGPTL1 mRNA expression was down-regulated in CRC tissues, and high ANGPTL1 expression predicted better survival in CRC patients. ANGPTL1 overexpression resulted in suppressed migration and invasion in vitro, and it prolonged overall survival in mouse models. By contrast, its down-regulation enhanced migration and invasion of CRC cells. MicroRNA-138 expression was positively correlated with ANGPTL1 mRNA level in CRC tissues and up-regulated by ANGPTL1 in CRC cells. In addition, the microRNA-138 inhibitor or mimics could reverse or promote the ANGPTL1-mediated inhibition of the migratory capacity of CRC cells, respectively.

**Conclusions:**

This study is the first to demonstrate the biological function of ANGPTL1 in CRC cells. ANGPTL1 expression was down-regulated in CRC tissues and inversely correlated with poor survival. ANGPTL1 repressed migration and invasion of CRC cells, and microRNA-138 was involved in this process.

**Electronic supplementary material:**

The online version of this article (doi:10.1186/s13046-017-0548-7) contains supplementary material, which is available to authorized users.

## Background

As the fourth most common cause of cancer-related deaths globally [1], colorectal cancer (CRC) is a major public health burden in most industrialized countries [2]. The poor efficiency and lack of options for treating metastasis is the major cause of death due to CRC. Specifically, the 5-year survival rate is 90.3% for patients with local disease, whereas it declines to 70.4 and 12.5% for patients with regional and distant metastasis, respectively [3]. Although great achievements have been made in medical science and technology over the last few decades, the understanding of the mechanisms underlying CRC development is still limited. It is imperative to explore the molecular events in CRC metastasis, which remains as one of the most difficult challenges encountered by modern oncologists.

In our previous study [4], we analyzed the gene expression profiles of paired tumor and normal tissue samples from The Cancer Genome Atlas (TCGA) datasets, and identified a number of genes that are significantly up-regulated or down-regulated in different types of cancer compared to their normal counterparts. Among them, we focused on Angiopoietin-like protein 1 (ANGPTL1), which was down-regulated in 87.5% (14/16) of included cancer types. ANGPTLs are a family of proteins that are structurally similar to angiopoietins, comprising ANGPTL1 to ANGPTL7. Members of this family contain a coiled-coil domain and a fibrinogen-like domain and are able to regulate angiogenesis. However, they do not bind to the receptors classically targeted by angiopoietins and are orphan ligands with no known receptors. More recent studies have proposed that they are involved in various pathologies, such as disorders of lipid and glucose metabolism, inflammation, hematopoiesis, and cancer [5, 6].

An early study showed that low expression of ANGPTL1 in lung and breast cancer tissues correlated with advanced-stage, higher grade tumor and lymph node status and poorer prognosis [7]. Further investigation revealed that ANGPTL1 suppressed SLUG-dependent epithelial-mesenchymal transition (EMT), thereby suppressing migratory and invasive capabilities of lung and breast cancer cell lines [7]. In addition, ANGPTL1 has been reported to inhibit the proliferation, migration, tube formation and adhesion of endothelial cells by blocking the MAPK and PI3K/Akt signaling pathways [8, 9]. Taken together, these findings suggest that ANGPTL1 may act as a novel tumor suppressor candidate in lung and breast cancer. However, its effects on CRC cells remain poorly defined.

Therefore, in this study, we explored the expression of ANGPTL1 in CRC specimens and paired normal tissues to gain a better understanding of its biological role in CRC. We found that ANGPTL1 was down-regulated in CRC tissues, and its low expression indicated shorter survival. In vitro and in vivo experiments showed that ANGPTL1 suppressed migration and invasion of CRC cells and prolonged overall survival (OS) in mouse models, which may be mediated by the up-regulation of microRNA-138 (miR-138). Our present study demonstrated for the first time that ANGPTL1 suppressed CRC metastasis and may be a novel target for the treatment of CRC.

## Methods

### Mining of differentially expressed genes

Expression data for ANGPTL1 in CRC and additional cancer types were extracted from level 3 TCGA RNA-seq data, totaling 705 paired tumor and normal samples. To determine the key differentially regulated genes between paired cancer and normal tissues, we compared gene expression profiles between cancer and normal groups by DEGSeq package for R/Bioconductor, and the *P* value was adjusted according to the false discovery rate. In addition, the relationship between the ANGPTL1 expression and its clinical manifestations was validated by publicly available independent microarray datasets (GSE32323 and GSE24550).

Furthermore, the GSE29623 and GSE35982 datasets, with information on both mRNA and microRNA (miRNA), were used to identify differentially expressed miRNA between high-ANGPTL1 and low-ANGPTL1 groups. All expression profiling data in this study were downloaded from TCGA (http://cancergenome.nih.gov/) and the Gene Expression Omnibus (GEO) (http://www.ncbi.nlm.nih.gov/geo/). We were able to use these databases by meeting the freedom-to-publish criteria of TCGA and NCBI.

### Patients and specimens

Tumor tissue samples and paired normal mucosal tissue were obtained by surgical resection and stored at −80 °C from 2009 to 2014 at the Second Affiliated Hospital of Zhejiang University, School of Medicine. Two pathologists confirmed these tissue samples as colorectal adenocarcinoma. This project was approved by the ethical committee of the Second Affiliated Hospital of Zhejiang University, School of Medicine and informed consent was obtained from all patients.

### Cell culture and reagents

All cells were cultured in RPMI-1640 (Gibco, Carlsbad, CA, USA) containing 10% fetal bovine serum (Life Technologies, Carlsbad, CA, USA) at 37 °C in a humidified atmosphere with 5% CO_2_. All cell lines were obtained from the American Type Culture Collection (Rockville, MD, USA). The lentiviral particles containing shRNA directed against ANGPTL1 (sc-88171-V) and corresponding scramble control (sc-108080) were purchased from Santa Cruz Technologies (Santa Cruz, CA, USA), and lentivirus containing firefly luciferase was purchased from Hanbio Biotechnology (Shanghai, China). miR-138 inhibitor, mimics and negative controls were synthesized by GenePharma (Shanghai, China) and were dissolved in DEPC-treated H_2_O.

### Lentivirus production and infection

The lentiviral vectors for ANGPTL1 were purchased from Cyagen Biosciences (Guangzhou, China), including pLV (Exp)-Puro-CMV > hANGPTL1/HA-IRES-eGFP and its control vector, pLV (Exp)-Puro-CMV > IRES-eGFP. One night prior to transfection, 293 T cells were plated in DMEM (Gibco) supplemented with 10% FBS without antibiotics. On the day of infection, the cells were transfected with a mixture of ANGPTL1 expression lentivector and pLV/helper packaging plasmids mix using Lipofectamine 2000 (Invitrogen, Carlsbad, CA, USA). The medium was replaced after overnight transfection. Supernatants were collected at 48 h post transfection, and filtered through 0.45 μm filters to remove cells and debris. Thus, the lentiviruses containing ANGPTL1/HA cDNA and the corresponding scramble control were harvested [10].

For lentiviral infection, cells were plated at 60–70% confluence. On the second day, the culture medium was replaced with complete medium containing appropriate lentiviral particles (MOI = 20) and Polybrene (2–5 μg/ml). Following 24 h of infection at 37 °C, the viral supernatant was replaced with fresh media. Another 48 h later, the infected cells were treated with 2.0 μg/ml puromycin dihydrochloride (Santa Cruz) for 2 weeks for selection of stable clones. The overexpression and knockdown efficiency was determined by quantitative real-time PCR (qPCR) and western blot (WB) analyses.

### Transfection of miRNA inhibitor or mimics

We transfected cells with a miRNA inhibitor or mimics using Lipofectamine 2000 according to the manufacturer’s instructions. Cells were seeded in 6-well plates and allowed to reach 60–70% confluence prior to transfection. The final concentration of the miR-138 inhibitor or mimics and their corresponding negative controls was 50 nmol/l. Twenty-four hours later, cells were harvested to evaluate the transfection efficiency. Then, successfully transfected cells were used for the following experiments.

For miR-138 inhibitor, the single-stranded RNA sequence was 5′-CGGCCUGAUUCACAACACCAGCU-3′. 5′- CAGUACUUUUGUGUAGUACAA-3′ was the sequence of its corresponding negative control. For miR-138 mimics, the sequences of oligonucleotides were 5′-AGCUGGUGUUGUGAAUCAGGCCG-3′ (sense), and 5′-GCCUGAUUCACAACACCAGCUUU-3′(antisense). And the sequences were 5′-UUCUCCGAACGUGUCACGUTT -3′(sense) and 5′- ACGUGACACGUUCGGAGAATT-3′ (antisense) for its negative control.

### qPCR

Total RNA from cells and fresh human tissues was isolated using RNAiso reagent (Takara Biotechnology, Dalian, China) according to the manufacturer’s instructions. The quality and quantity of RNA were evaluated using NanoDrop 1000 spectrophotometer (Thermo Scientific, Pittsburgh, PA, USA). cDNA was synthesized with PrimeScript™ II 1st Strand cDNA Synthesis Kit (Takara Biotechnology). To validate the mRNA expression profiles, qPCR was performed using a standard SYBR-Green PCR kit protocol (Takara Biotechnology) with the StepOne Plus Real Time PCR System (Life Technologies). The primers were synthesized by Sangon Biotech (Shanghai, China), and the sequences were as follows: ANGPTL1 forward: 5′-CAACATATTCCTAACAGCCAACAG -3′, reverse: 5′-TGACAGTCTTTGAATGGTCCTTC -3′; GAPDH forward: 5′- TCTCTGCTCCTCCTGTTCGA -3′, reverse: 5′- GCGCCCAATACGACCAAATC -3′. All PCR reactions were performed in triplicate. GAPDH was used as an internal control.

For quantifying mature miR-138, reverse transcription was performed using a miRNA 1st Strand cDNA Synthesis kit (Sangon Biotech, Shanghai, China) according to the manufacturer’s protocol. The reverse transcription primer for miR-138 was 5′-GTCGTATCCAGTGCAGGGTCCGAGGTATTCGCACTGGATACGACCGGCCT-3′, and primer for small nuclear RNA U6 was 5′- GTCGTATCCAGTGCAGGGTCCGAGGTATTCGCACTGGATACGACAAAATA-3′. The mature miR-138 level was normalized with U6 determined by qPCR, as described previously. Primers sequences were as follows: miR-138 forward: 5′-AAGCGGAGCTGGTGTTGTGAATC-3′, reverse: 5′- ATCCAGTGCAGGGTCCGAGG-3′; U6 forward: 5′-AGAGAAGATTAG CATGGCCCCTG-3′, reverse: 5′-ATCCAGTGCAGGGTCCGAGG-3′.

### WB analysis

WB analysis was performed as described previously [11]. Briefly, cell protein was extracted using Mammalian Protein Extraction Reagent (Thermo Scientific, Pittsburgh, PA, USA) supplemented with 1% protease inhibitor cocktails (Sigma-Aldrich, Hamburg, Germany). Protein concentration was measured using a BCA protein assay kit (Thermo Scientific). The protein samples (10–20 μg) were separated by 12% SDS-PAGE, transferred to a PVDF membrane (Bio-Rad, Hercules, CA, USA) and then detected with appropriate primary and secondary antibodies. Protein bands were visualized by chemiluminescence (Thermo Scientific) and scanned via a Kodak Image Station (Carestream Health, Inc., Rochester, New York, USA). The primary antibodies used were goat anti-ANGPTL1 polyclonal antibody (1:1000, R&D Systems, Minneapolis, MN, USA) and rabbit anti-GAPDH monoclonal antibody (1:1000, Cell Signaling Technology, Beverly, MA, USA).

### Transwell migration and invasion assay

Cells resuspended in 200 μl serum-free medium were seeded in the upper chamber with 10% serum-containing medium in the lower chamber of 24-well transwell plates (Corning Inc., NY, USA). After 48 or 72 h, the non-invaded cells in the upper chamber were removed with cotton swabs, and HE staining solution was then used to stain the invaded cells. Images were taken at 10× or 20× magnification. In addition, cell numbers were counted in at least 5 random microscope fields. Matrigel-coated 8 μm-pore transwells (Corning Inc.) were used for the invasion assay. The procedures and analyses were the same as those for the transwell migration assay except for the presence of Matrigel.

### Cell proliferation assay

Cell proliferation was analyzed using the Cell Counting Kit-8 (CCK-8) (Dojindo Laboratories, Tokyo, Japan). Cells were seeded into 96-well plates at the density of 5 × 10^3^ cells/200 μl per well. 20 μl CCK-8 solution will be added to cells in culture and incubated for 1 h. The absorbance of each well was measured using a microculture plate reader at a wavelength of 450 nm. Three replicate wells were set up in each group and three independent experiments were performed, respectively.

### Colony-forming assay

Five hundred cells were inoculated into the 6-well plate containing 3 mL medium, which was changed every 3 days. After 2 weeks, clone spheres were formed. Cells were rinsed with PBS, and then fixed with 4% paraformaldehyde for 15 min. Crystal violet was added for staining for 15 min, and the plates were rinsed with flow water, and then air-dried. The number of clones in each plate was counted.

### Mice

Balb/c athymic nude mice (SLAC Laboratory Animal Co. Ltd., Shanghai, China) were maintained and subjected to the experiments in accordance with the protocols approved by the Second Affiliated Hospital of Zhejiang University School of Medicine Animal Care and Use Committee. All animal experiments were performed on 5 to 6 weeks old female Balb/c athymic nude mice.

### Subcutaneously inoculated model

Tumor cells (1 × 10^6^) were injected subcutaneously into mice. Three weeks after injection, the mice were euthanized, and the tumors were excised. Long (L) and short (S) axes of each tumor were measured with calipers. Tumor volume (V) was calculated as follows: V = (LxS^2^)/2. In addition, tumor weight was also measured.

### Hemi-spleen liver metastasis model

The CRC hemi-spleen liver metastasis model was established by using a previously described technique [12]. Mice were anaesthetized and an incision was made between the left abdominal and thoracic regions, and the spleen was exposed. The spleens were divided into two halves, and the halves were clipped. In total, 2 × 10^6^ cells were injected into the splenic vessels (splenic artery and veins) through one hemi-spleen followed by flushing with PBS. After the injection, the splenic vessels draining the injected hemi-spleen were clipped, and the hemi-spleen was removed. The abdominal wall and skin were sutured. Tumor metastasis was monitored using a small animal IVIS Lumina imaging system (Caliper Life Sciences, Hopkinton, MA) 15 min after intraperitoneal administration of D-luciferin (PekinElmer Inc., Boston, USA) at a concentration of 150 mg/kg. All mice were kept until death due to the neoplastic process or until the end of the experiment (2 months), and the livers were harvested for histological analysis of metastasis.

### Orthotopic injection model

In brief, mice were anesthetized, and the cecum was exteriorized by laparotomy. A total volume of 50 μl cell suspension containing 5x10^6^ tumor cells was injected into the cecal wall by a 26G needle. Then, the injection point was slightly pressed with a cotton stick and inspected to ensure that there was no leakage. Afterward, the cecum was returned to the abdominal cavity, and the skin was closed with running sutures [13]. All mice were kept until death due to the neoplastic process or until the end of the experiment (3 months). Livers were harvested for histological analysis of metastasis.

### Statistical analysis

All graphing and statistical analyses were performed using GraphPad Prism version 6.0 (GraphPad Software, La Jolla, CA, USA). The data are presented as the means ± standard errors of the mean. The gene expression and qPCR results from paired clinical samples were analyzed by two-tail paired Student’s t-test. The comparison of survival between groups was performed using the log-rank test, and Kaplan-Meier curves were plotted. The other results were analyzed by two-tail unpaired Student’s t-test. Pearson correlation analysis was used to measure the relationship between ANGPTL1 and miR-138. *P* values <0.05 indicated statistical significance.

## Results

### ANGPTL1 mRNA expression was down-regulated in CRC, and high ANGPTL1 expression correlated with better survival

**Fig. 1:**
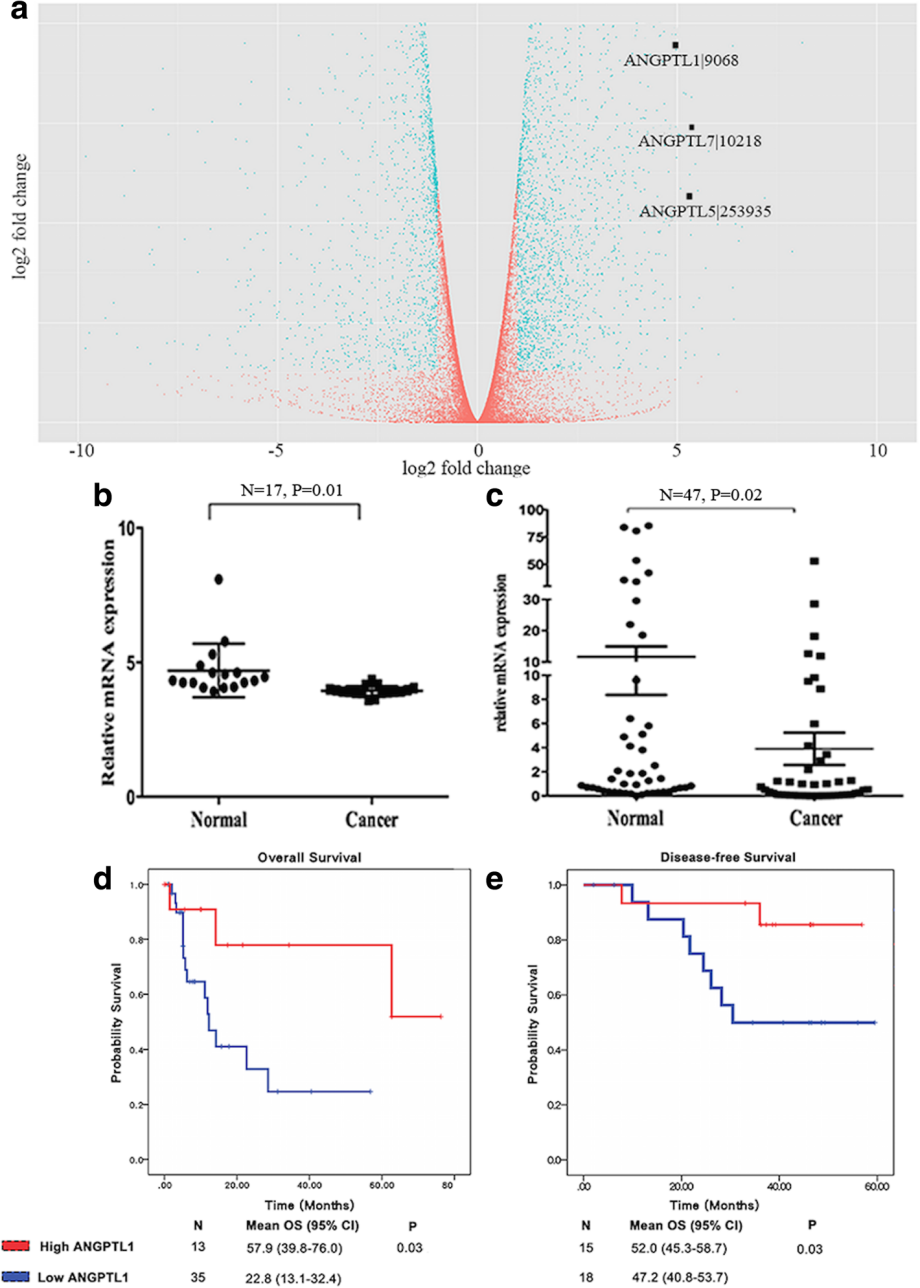
ANGPTL1 mRNA expression is down-regulated in CRC tissues, and its high expression predicts better survival. **a** Volcano plot of the gene expression data from TCGA. The x-axis denotes the log2 fold change of gene expression in normal relative to tumor tissues (i.e., 5 represents a log2 fold change of 5.0 in normal vs. tumor tissues). Genes represented with *blue dots* are highly statistically significant with greater than 2-fold changes. The results demonstrated that ANGPTL1, ANGPTL5 and ANGPTL7 were significantly down-regulated in CRC samples. **b** In the GSE32323 dataset, ANGPTL1 was expressed at low levels in tumor samples (*P* = 0.01). **c** Similar reduction in the expression of ANGPTL1 in CRC was also found in patients at our oncology center (*P* = 0.02). **d** Patients with high ANGPTL1 expression in TCGA dataset had significantly longer OS than those with low levels of ANGPTL1 expression in stage IV CRC patients (*P* = 0.03). **e** In the GSE24550 cohort, high expression of ANGPTL1 predicted better disease-free survival in stage III patients (*P* = 0.03)

To identify genes of tumorigenic potential, we analyzed gene expression profiles of paired cancerous and normal tissues from TCGA datasets. Gene expression analyses revealed that ANGPTL1, ANGPTL5 and ANGPTL7 were significantly down-regulated in CRC samples (Fig. 1a). Among them, ANGPTL1 was most significantly changed, and, remarkably, it was down-regulated in 87.5% (14/16) of cancer types, including breast invasive carcinoma, lung adenocarcinoma and thyroid carcinoma (Table 1). Apart from ANGPTL1, most ANGPTLs were also dramatically down-regulated in tumor tissues compared to the corresponding normal samples (Additional file 1: Table S1). Next, we validated our finding in the GSE32323 cohort from the GEO database. Analysis of 17 paired CRC and normal samples showed that ANGPTL1 was down-regulated in tumor samples (*P* = 0.01) (Fig. 1b). Finally, we confirmed these results in our oncology center. The basic characteristics of these patients are specified in Additional file 2: Table S2. Unsurprisingly, the results were similar to what we had found in TCGA and GEO datasets (*P* = 0.02, Fig. 1c). The low expression of ANGPTL1 in CRC and many other cancer types suggested that it was a potential suppressor of cancer and may be associated with CRC initiation and progression.

**Table 1:** ANGPTL1 expression was significantly down regulated in cancer tissues of 14 cancer types

Cancer Type	Number^a^	Normal^b^	Cancer^b^	log2FoldChange^c^	Adjusted *P* value^d^
BLCA	19	637.89	25.25	−4.66	<0.0001
BRCA	113	600.86	74.84	−3.01	<0.0001
CHOL	9	427.26	107.93	−1.98	<0.0001
CRC	32	797.1	38.74	−4.36	<0.0001
ESCA	11	816.82	40.15	−4.35	0.04
HNSC	43	418.62	31.68	−3.72	<0.0001
KIRC	72	529.9	63.99	−3.05	<0.0001
KIRP	72	439.06	80.07	−2.46	<0.0001
LUAD	58	300.09	44.4	−2.76	<0.0001
LUSC	51	309.17	27.88	−3.47	<0.0001
PRAD	52	287.12	81.15	−1.82	<0.0001
STAD	32	1520.86	154.21	−3.3	<0.0001
THCA	59	1423.74	382.68	−1.9	<0.0001
UCEC	7	1267.48	87.89	−3.85	<0.0001

*Abbreviations*: *BLCA* bladder urothelial carcinoma, *BRCA* breast invasive carcinoma, *CHOL* cholangiocarcinoma, *CRC* colorectal cancer, *ESCA* esophageal carcinoma, *HNSC* head and neck squamous cell carcinoma, *KIRC* kidney renal clear cell carcinoma, *KIRP* kidney renal papillary cell carcinoma, *LUAD* lung adenocarcinoma, *LUSC* lung squamous cell carcinoma, *PRAD* prostate adenocarcinoma, *STAD* stomach adenocarcinoma, *THCA* thyroid carcinoma, *UCEC* uterine corpus endometrial carcinoma

^a^Number of paired normal and cancer tissues included in this study. We only considered the cancer type with six or more samples for differential expression analysis

^b^Mean expression level of normal or cancer tissue

^c^Fold change is calculated by mean expression level of Cancer/Normal

^d^ *P* value was performed by DEGSeq package for R/Bioconductor and adjusted *P* value was counducted according to false discovery rate

To further determine the clinical significance of ANGPTL1 expression, we explored its association with survival information in TCGA dataset. The results showed that patients with high ANGPTL1 mRNA level had significantly longer OS than patients with low level of ANGPTL1 in stage IV metastatic CRC (*P* = 0.03, Fig. 1d). Moreover, in the GSE24550 cohort, high expression of ANGPTL1 predicted better disease-free survival in stage III patients (*P* = 0.03, Fig. 1e). Taken together, these results suggested that high ANGPTL1 expression was correlated with better survival in CRC patients.

### ANGPTL1 exhibits no significant effects on proliferative and colony-forming capacity

To investigate the little-known function of ANGPTL1 in the biological behavior of CRC cells, ANGPTL1-targeting shRNA or ANGPTL1 cDNA plasmids packaged in lentiviruses were used to modulate ANGPTL1 gene expression in human CRC cell lines SW480 and SW620. Overexpression and knockdown efficiency of ANGPTL1 was confirmed by qPCR and WB (Additional file 3: Figure S1A). At first, we assessed the effect of ANGPTL1 on tumor cell proliferation using the CCK-8 kit, and the results showed no significant difference between SW620-ANGPTL1 and SW620-Ctrl cells (Additional file 3: Figure S1B, *P* = 0.10). No significant difference was found by colony-forming assay as well (Additional file 3: Figure S1C, *P* = 0.27). Similar results were found in SW480-shANGPTL1 and SW480-Ctrl cells (Additional file 3: Figure S1D, E).

To further confirm our findings, we compared the growth of SW620-ANGPTL1 and SW620-Ctrl cells in a subcutaneously inoculated model. Cells were injected subcutaneously into the flanks of nude mice (*N* = 5 for each group). Three weeks after injection, tumor weight (*P* = 0.29) and volume (*P* = 0.53) showed no significant differences between SW620-ANGPTL1 and SW620-Ctrl group (Additional file 3: Figure S1F-H). Taken together, these results indicated that ANGPTL1 does not significantly affect proliferation and colony formation of CRC cells.

### ANGPTL1 represses migration and invasion of CRC cells in vitro

**Fig. 2:**
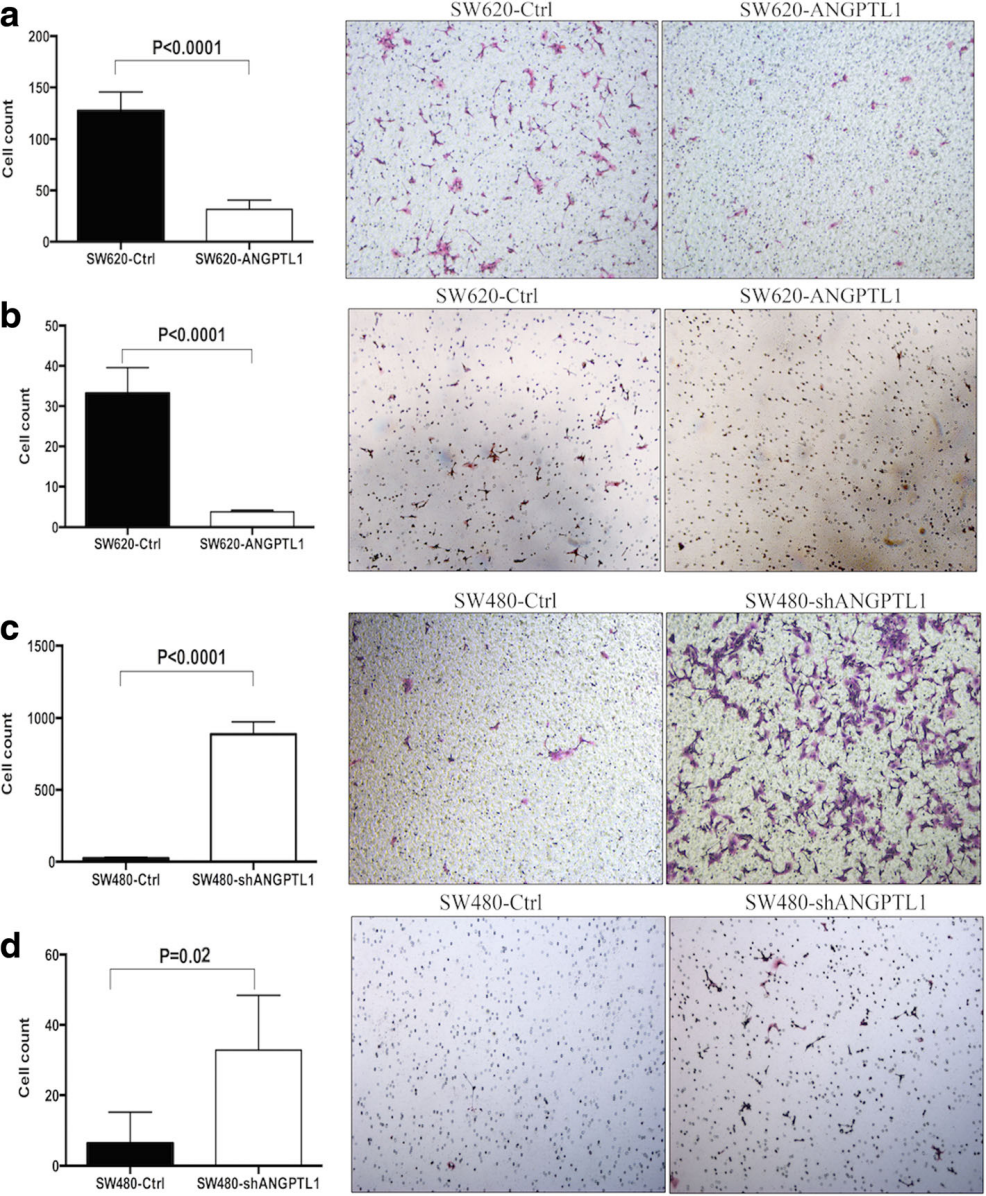
ANGPTL1 represses migration and invasion of CRC cells in vitro. **a** SW620-ANGPTL1 cells exhibited significantly reduced mobility compared to vector control cells (*P* < 0.0001). **b** Invasion assay indicated that SW620-ANGPTL1 cells had significantly reduced invasion capacity compared to SW620-Ctrl cells (*P* < 0.0001). **c**-**d** The number of invading cells was significantly higher in the SW480-shANGPTL1 cells compared to that in the SW480-Ctrl cells by transwell migration (*P* < 0.0001) (**c**) and invasion assay (*P* = 0.0159) (**d**)

Next, we examined the role of ANGPTL1 in migration and invasion by transwell assay. As shown in Fig. 2a, SW620-ANGPTL1 cells exhibited remarkably reduced mobility compared to vector control cells (*P* < 0.0001). Matrigel-coated invasion assay also indicated that SW620-ANGPTL1 cells displayed significantly decreased invasion capacity compared to SW620-Ctrl cells (*P* < 0.0001) (Fig. 2b). By contrast, the number of invading cells was significantly increased in SW480-shANGPTL1 cells compared to that in SW480-Ctrl cells in both transwell migration (*P* < 0.0001, Fig. 2c) and invasion assay (*P* = 0.02, Fig. 2d). In addition, knockdown of ANGPTL1 in LoVo cells produced the same results as in the SW480 cells (Additional file 4: Figure S2). It can be inferred that the decrease in the number of invading cells by transwell assay is due to the effect of ANGPTL1 on migration and invasion rather than on proliferation, because ANGPTL1 has no significant effects on cell proliferation. Collectively, these results indicated that ANGPTL1 represses migration and invasion of CRC cells in vitro.

### ANGPTL1 inhibited liver metastasis of CRC and prolonged OS in vivo

The liver is the most common site of metastasis in CRC. To investigate the effect of ANGPTL1 on the metastatic potential of CRC cells, we employed a hemi-spleen liver metastasis model, in which tumor cells suspensions were injected into a part of the spleen, with successive hemi-splenectomy. The other half of the spleen, uncontaminated with tumor cells, was retained in the animal. As the tumor cells were inoculated into the portal vein, diffuse liver metastasis was established [14, 15]. Additionally, the orthotopic injection model, with cancer cells injected into the cecum for local tumor growth, better mimics the natural trajectory of colon cancer in the patients [16]. Therefore, this model was also used to further evaluate spontaneous formation of liver metastasis.

**Fig. 3:**
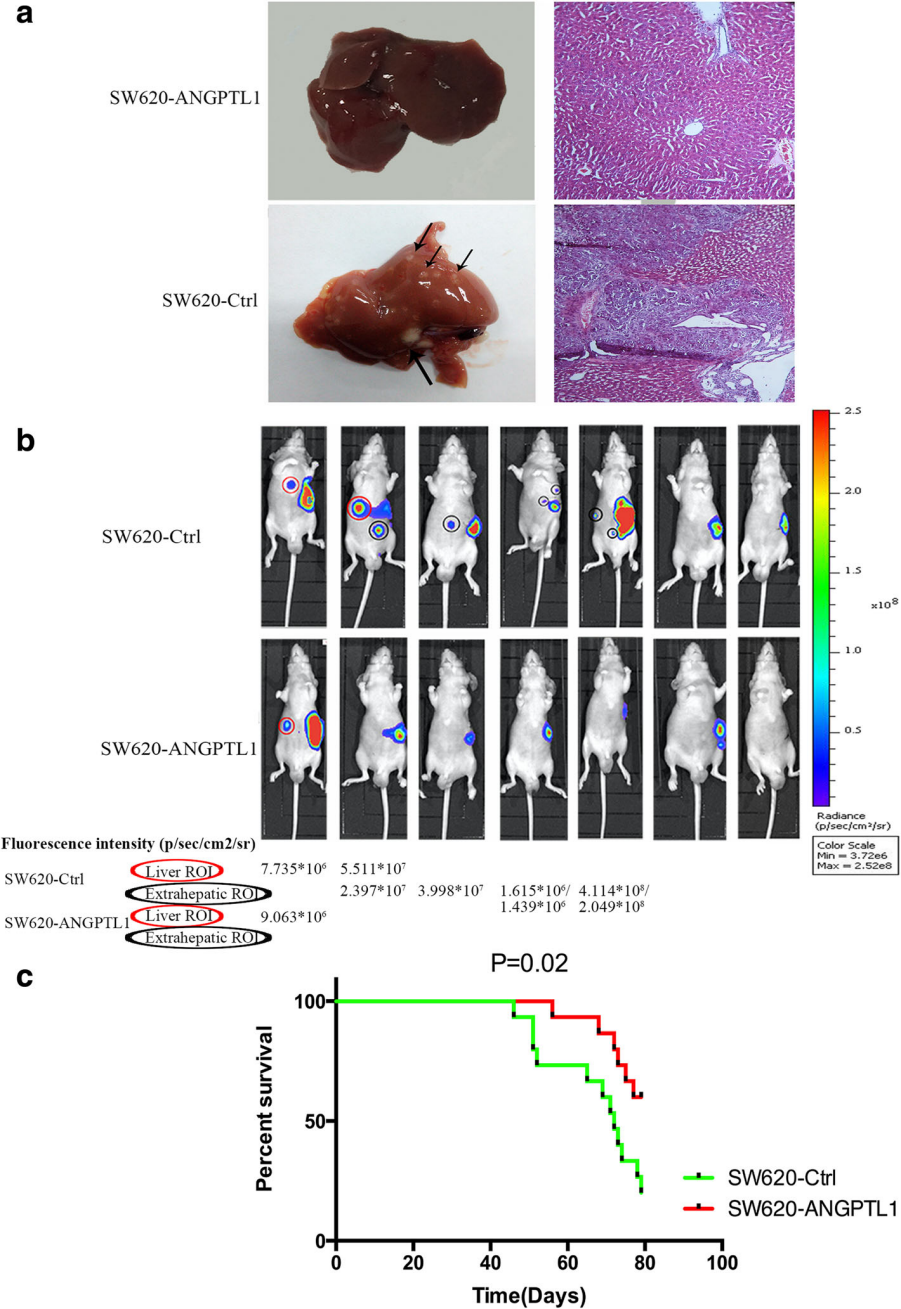
ANGPTL1 inhibits liver metastasis in CRC and prolongs OS in vivo. **a** Histological examination of liver sections from SW620-ANGPTL1 and SW620-Ctrl groups. Liver lesions (*black arrow*) were confirmed as liver metastasis of CRC. **b** In the hemi-spleen liver metastasis model, the SW620-ANGPTL1 group had lower incidence of liver metastasis (*red circle*) and lower radiance intensity of liver lesions than the SW620-Ctrl group, which was illustrated by the in vivo imaging system. In addition, the rate of extrahepatic metastasis (*black circle*) was also lower in SW620-ANGPTL1 cells. **c** The mice in the SW620-ANGPTL1 group had longer OS (*P* = 0.02)

In the hemi-spleen liver metastasis model, mice were sacrificed 2 months after injection of tumor cells, and livers were harvested for histological examination of metastasis (Fig. 3a). We found that the incidence of liver metastasis was lower in the SW620-ANGPTL1 group compared to that in the SW620-Ctrl group (14.29% vs 28.57%) (Table 2). Moreover, we used the IVIS Lumina imaging system to monitor the migration of tumor cells in vivo on day 14. The fluorescence intensity of liver lesions was higher in the SW620-Ctrl group (Fig. 3b). Apart from liver metastasis, we found that the rate of extrahepatic metastasis in SW620-ANGPTL1 cells was also lower than that in SW620-Ctrl cells using the in vivo imaging system (Fig. 3b). In addition, the mice in this group displayed longer OS (*P* = 0.02, Fig. 3c). Similarly, in the orthotopic injection model, the rate of liver metastasis was decreased in the SW620-ANGPTL1 group (14.29% vs, 28.57%) (Table 2), which was confirmed by histological analysis.

**Table 2:** Liver metastasis rate of different cell lines

Liver metastasis model	Group	Total No. of mice	Total No. of mice with metastasis	Rate
Orthotopic injection model	SW620-Ctrl	7	2	28.57%
	SW620-ANGPTL1	7	1	14.29%
	SW480-Ctrl	18	2	11.11%
	SW480-shANGPTL1	20	6	30.00%
Hemi-spleen injection model	SW620-Ctrl	7	2	28.57%
	SW620-ANGPTL1	7	1	14.29%
	SW480-Ctrl	16	1	0.06%
	SW480-shANGPTL1	19	4	21.05%

By contrast, the incidence of liver metastasis in the SW480-shANGPTL1 group increased to 21.05%, whereas the control group had a very low rate of liver metastasis (0.06%) in the hemi-spleen liver metastasis model (Table 2). Additionally, in the orthotopic injection model, the rate of liver metastasis was higher in the SW480-shANGPTL1 group compared to that in the control (30.00% vs 11.11%). Collectively, high ANGPTL1 expression level is associated with a reduction in CRC metastasis and better prognosis in mice with CRC.

### miR-138 was up-regulated by ANGPTL1 and involved in ANGPTL1-mediated inhibition of migration of CRC cells

**Fig. 4:**
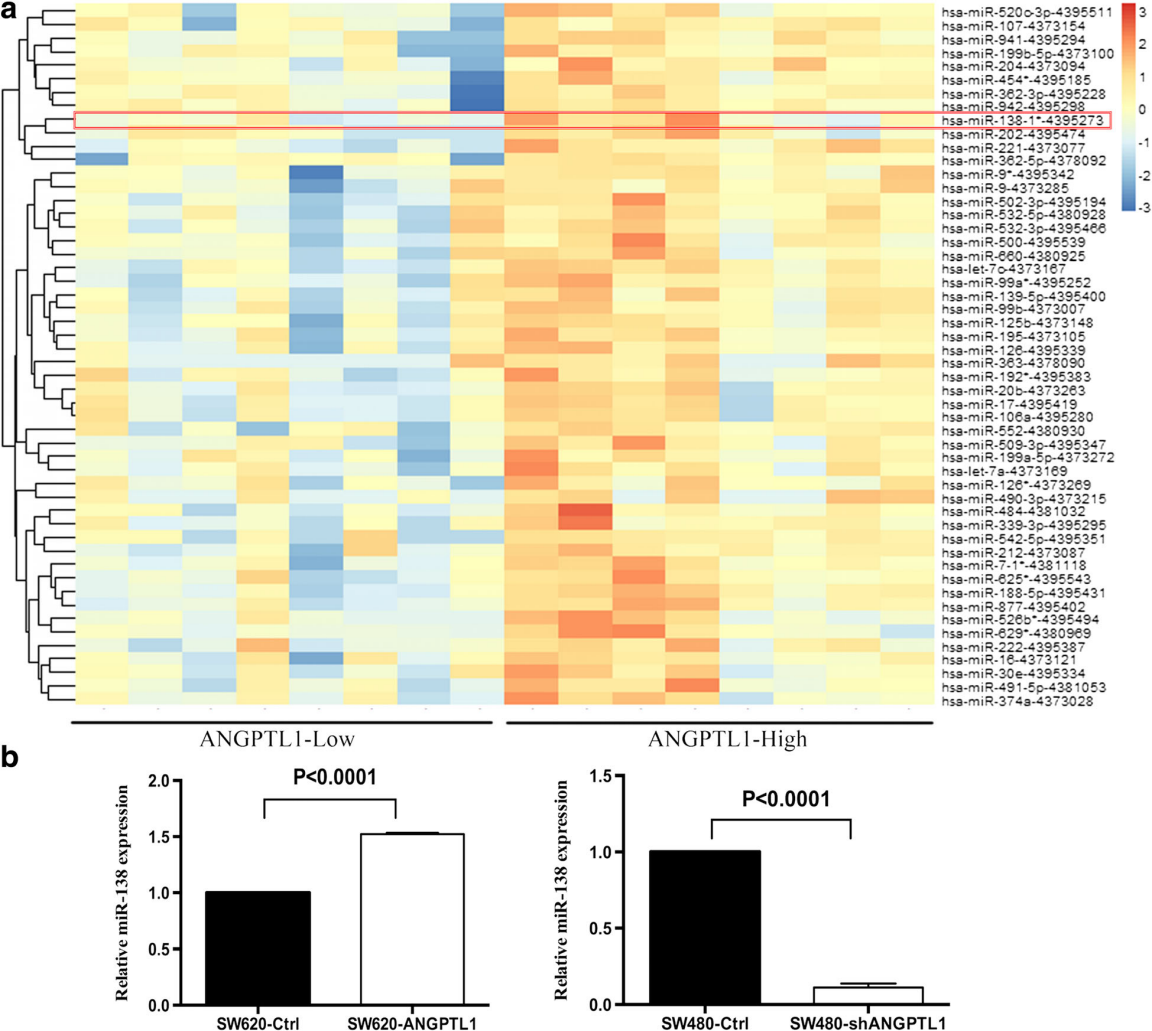
**a** Heatmap illustrating the diversity of miRNA levels among 8 samples with the lowest ANGPTL1 mRNA level and 8 samples with the highest ANGPTL1 mRNA level. Among the miRNAs, the level of miR-138 was significantly higher in the samples with high levels of ANGPTL1 (*P* = 0.01). **b** Level of miR-138 in cells with overexpression and knockdown of ANGPTL1. In SW620-ANGPTL1 cells, the expression of miR-138 was significantly enhanced compared to control cells (*P* < 0.0001), while it was markedly inhibited in SW480-shANGPTL1 cells (*P* < 0.0001)

Accumulating evidence has demonstrated critical roles for miRNAs in various cellular processes, including tumor development and progression. Kuo et al. [7] demonstrated that ANGPTL1 regulates the expression of miR-630 at the transcriptional level, which targets SLUG and results in the inhibition of lung cancer cell metastasis. Thus, we considered whether miRNAs are involved in the ANGPTL1-mediated inhibition of migration of CRC cells, as well as the specific miRNAs contributing to this process. To further verify this hypothesis, we extracted the profiling data for both miRNAs and mRNAs from the GSE29623 dataset to address the relationship between ANGPTL1 and miRNAs in CRC. The 8 samples with the lowest ANGPTL1 mRNA level were compared with the 8 samples with the highest ANGPTL1 mRNA level, and a heatmap was formulated to measure the diversity of miRNA level (Fig. 4a). Among these candidates, miR-138 was reported to be down-regulated in CRC tissues, and its down-regulation was associated with more severe metastasis in vitro and in vivo by targeting TWIST2 [17]. miR-138 was suggested to be of great priority of being involved in ANGPTL1-regulated metastasis. To validate this finding, we explored the association between transcript levels of ANGPTL1 and miR-138 in another GSE dataset (GSE35982). Pearson correlation analysis suggested that ANGPTL1 mRNA level was positively correlated with miR-138 level in CRC tumor samples (Pearson correlation value = 0.94, *P* = 0.001). However, no significant linear relationship was found in normal samples (Pearson correlation value = -0.45, *P* = 0.27). The specific data for expression levels of ANGPTL1 and miR-138 in tumor tissues are plotted in Additional file 5: Figure S3A.

**Fig. 5:**
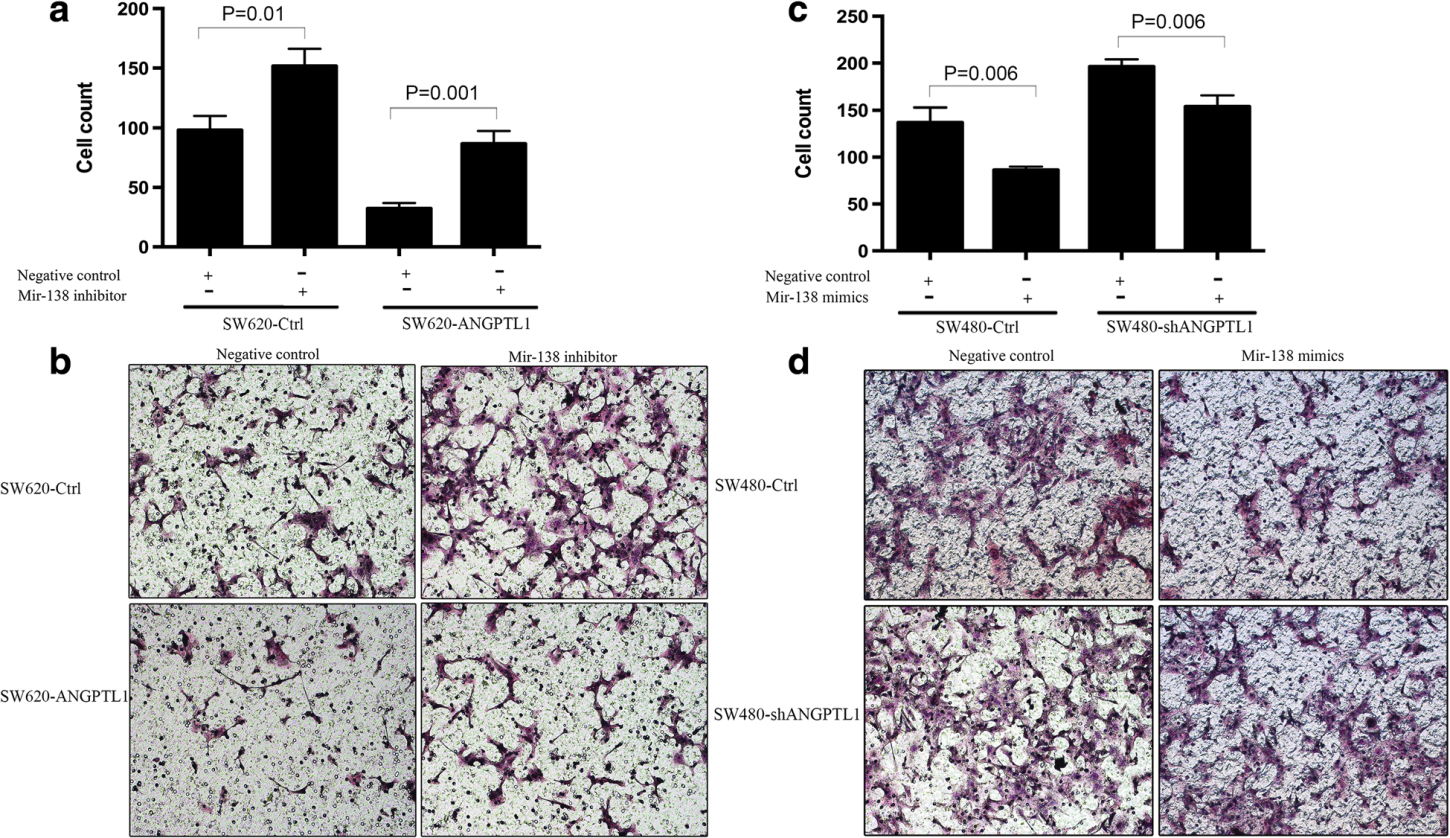
miR-138 was involved in the ANGPTL1-mediated inhibition of migration of CRC cells. **a** SW620-Ctrl cells treated with miR-138 inhibitor showed enhanced migratory capacity (*P* = 0.01), and miR-138 inhibitor reversed the inhibition of migration in SW620-ANGPTL1 cells (*P* = 0.001). **b** Representative images of transwell migration assay in cells treated with/without miR-138 inhibitor. **c** SW480-Ctrl cells treated with miR-138 mimics showed decreased migratory capacity (*P* = 0.006), and miR-138 mimics reversed the promotion of migration in SW480-shANGPTL1 cells (*P* = 0.006). **d** Representative images of transwell migration assay in cells treated with/without miR-138 mimics

The level of miR-138 was further determined in cells with ANGPTL1 overexpression and knockdown. As shown in Additional file 5: Figure S3B, overexpression of ANGPTL1 significantly enhanced miR-138 expression by 1.51-fold (*P* < 0.0001), whereas miR-138 level was markedly (86%) inhibited by knockdown of ANGPTL1 (*P* < 0.0001), indicating that ANGPTL1 may regulate the expression of miR-138. Then, we conducted transwell migration assay to determine whether miR-138 is involved in ANGPTL1-mediated inhibition of migration of CRC cells. Cells were transiently transfected with a miR-138 inhibitor, mimics or negative controls at 50 nmol/l using Lipofectamine 2000. As shown in Fig. 5a-b, SW620-Ctrl cells treated with miR-138 inhibitor showed enhanced migratory capacity (*P* = 0.01), and miR-138 inhibitor reversed the inhibition of migration in SW620-ANGPTL1 cells (*P* = 0.001). In addition, in SW480-shANGPTL1 cells transfected with miR-138 mimics, the shANGPTL1-induced increase in migratory capacity was significantly attenuated (*P* = 0.006, Fig. 5c-d). These findings suggest that ANGPTL1 directly or indirectly up-regulates the expression of miR-138, and miR-138 is involved in ANGPTL1-mediated inhibition of migration of CRC cells.

## Discussion

In this study, we compared the gene expression profiles of paired cancerous and normal tissues from TCGA datasets, and identified ANGPTL1 as a down-regulated gene in CRC. Further in vitro and in vivo studies confirmed that ANGPTL1 inhibited migration and invasion with limited effects on CRC cell proliferation and colony formation of CRC cells. Finally, we found that ANGPTL1 exerts its effect by up-regulating miR-138. This study is the first to investigate the role of ANGPTL1 in the biology and progression of CRC.

Similar to our results, previous studies have reported that ANGPTL1 was significantly decreased in lung [7, 18] and breast [7] tumor tissues compared to normal tissues. In addition, the inhibition of migration in CRC was consistent with the conclusions of a study by Kuo et al.[7], in which ANGPTL1 was reported to inhibit the migration and invasion of lung and breast cancer cells via mesenchymal-epithelial transition. Together, these studies congruously characterized ANGPTL1 as a tumor suppressor gene in cancer.

miRNAs are involved in post-transcriptional and translational silencing of target genes by binding to complementary sequences in 3’ UTRs [19]. Therefore, miRNAs are crucial in the regulation of many crucial biological processes, such as detachment, migration, invasion and colonization of cancer cells [20, 21]. It has been reported that important signaling pathways in CRC, such as the Wnt/β-catenin, RAS, p53, TGF-β, NF-kB pathways, are regulated by miRNAs [21–23]. A number of studies have demonstrated that miR-138 regulates various molecular pathways and is associated with initiation and progression of cancer, and thus is considered as a potential tumor suppressor [24]. As reported by Jiang et al.[25], ectopic transfection of miR-138 contributed to the reduced migration and invasion in oral tongue squamous cell carcinoma by targeting RhoC and ROCK2, which are involved in the remodeling of cellular cytoskeleton. In clear cell renal cell carcinoma cells, miR-138 reduced the expression of hypoxia-inducible factor-1 alpha, which in turn enhanced apoptosis and decreased cell migration [26]. In addition, miR-138 was down-regulated in CRC tissues, and this down-regulation was associated with more severe metastasis in vitro and in vivo by targeting TWIST2 [17]. Our results also confirmed that miR-138 expression is positively correlated with ANGPTL1 mRNA level in CRC tissues and is involved in ANGPTL1-induced attenuated migration of CRC cells, which was consistent with the reports of Long et al.[17].

The transcription of miRNAs is carried out by RNA Polymerase II and controlled by RNA Polymerase II-associated transcriptional factors and multiple epigenetic factors. Following transcription, the pri-miRNA undergoes several steps of maturation, including Drosha and Dicer processing in the nucleus, Exportin 5-mediated nuclear export, and cytoplasmic processing [19, 20]. Collectively, miRNAs are regulated at multiple levels. As reported by Kuo et al [7], ANGPTL1 up-regulates miR-630 expression at the transcriptional level, thus leading to increase of both pre-miR-630 and pri-miR-630 expression. In this study, we found that miR-138 was up-regulated by ANGPTL1, but the mechanism of its biogenesis remains unexplored. As reported, epigenetic and transcription factors, as well as many other molecules, are correlated with miR-138 expression [24]. For example, miR-138 expression can be decreased by methylation of its DNA [27] and up-regulated by a histone deacetylase inhibitor [28] and overexpression of P 19 H-Ras [29]. ANGPTL1 might directly or indirectly regulate miR-138 expression via the above mechanisms.

With respect to the potential targets of miR-138, EMT, the TGF-β pathway, the RhoC-Erk-MMP-2/9 pathway, and the cofilin pathway have been identified to participate in the reduced migratory and invasive activity induced by miR-138 [24]. Specifically, miR-138 overexpression inhibits EMT process by targeting Vimentin and EZH2, thus reducing breast cancer invasion [30]. In CRC patients, miR-138 targets TWIST2, a crucial regulator of EMT, to attenuate metastasis [17]. Because ANGPTL1 has also been reported to regulate EMT to attenuate metastasis [7], it is likely that EMT might be a potential mechanism in the ANGPTL1-miR-138-induced inhibition of metastasis in CRC, which requires further exploration.

In summary, we provide clinical evidence that ANGPTL1 expression is down-regulated in CRC tissues and inversely correlated with survival in patients with cancer. We demonstrate that ANGPTL1 represses migration and invasion of CRC cells by up-regulating miR-138. Future studies are warranted to investigate the underlying mechanisms by which ANGPTL1 regulates the transcription of miR-138, and the target genes that are involved in the ANGPTL1-miR-138-induced inhibition of metastasis in CRC. Such studies will provide more insights into CRC and provide a rationale for the utilization of innovative therapy in targeting ANGPTL1 to improve CRC treatment.

## Conclusion

ANGPTL1 expression was down-regulated in CRC tissues and inversely correlated with poor survival. ANGPTL1 repressed migration and invasion of CRC cells, and that miR-138 was involved in this process.

## Additional files

Additional file 1: Table S1. ANGPTL2-7 expression differences between normal and cancer tissues in different types of cancers. (DOCX 83 kb)

Additional file 2: Table S2. Basic characteristics of CRC patients in our center. (DOCX 35 kb)

Additional file 3: Figure S1. ANGPTL1 does not affect the proliferative and colony-forming capacity of CRC cells. A. Generation of ANGPTL1-overexpressing/knockdown cell lines. SW620-ANGPTL1 cells exhibited an increase in ANGPTL1 at mRNA and protein levels, whereas ANGPTL1 expression was decreased in SW480-shANGPTL1 cells. B. Cell proliferation assay showed no significant difference between SW620-ANGPTL1 and SW620-Ctrl cells (*P* = 0.10). C. No significant difference was found by colony formation assay as well (*P* = 0.27). D. Cell proliferation assay showed no significant difference between SW480-shANGPTL1 and SW480-Ctrl cells (*P* = 0.22). E. Colony formation assay also revealed no significant difference between these two groups (*P* = 0.54). F. Subcutaneous tumor formed by SW620-ANGPTL1 and SW620-Ctrl cells. G-H: Tumor weight (*P* = 0.29) (G) and volume (*P* = 0.53) (H) were not significantly different between these two groups. (PNG 702 kb)

Additional file 4: Figure S2. ANGPTL1 knockdown promotes migration and invasion in LoVo cells. A. The number of invading cells was significantly higher in the LoVo-shANGPTL1 cells compared to that in the LoVo-Ctrl cells by transwell migration (*P* = 0.04) (A) and invasion assay (*P* = 0.0005) (B). (PNG 2705 kb)

Additional file 5: Figure S3. A. Expression data of ANGPTL1 and miR-138 in 8 CRC tumor tissues from patients extracted from the GSE35982 dataset. The levels of ANGPTL1 and miR-138 were positively correlated (Pearson correlation value = 0.94, *P* = 0.001). B. miR-138 inhibitor or mimics decreased or enhanced the expression of miR-138, respectively, whereas the negative controls had no significant effects on its expression. (PNG 2675 kb)

## Abbreviations

ANGPTL1: Angiopoietin-like protein 1
CCK-8: Cell counting Kit-8
CRC: Colorectal cancer
EMT: Epithelial mesenchymal transition
GEO: Gene expression omnibus
L: Long
miR-138: MicroRNA-138
miRNA: MicroRNA
OS: Overall survival
qPCR: Quantitative real-time PCR
S: Short
TCGA: The Cancer Genome Atlas
V: Tumor volume
WB: Western blot
