# Supplementary material for: ANGPTL1 attenuates colorectal cancer metastasis by up-regulating microRNA-138
Source: J Exp Clin Cancer Res. 2017 Jun 12;36:78. doi: 10.1186/s13046-017-0548-7 (PMC5467265; doi:10.1186/s13046-017-0548-7)
Supplement: Supplementary file 1 — ANGPTL2-7 expression differences between normal and cancer tissues in different types of cancers. (DOCX 83 kb) [file 13046_2017_548_MOESM1_ESM.docx]

| **Table S1. ANGPTL2-7 expression differences between normal and cancer tissues in different types of cancers.** | | | | | | |
| --- | --- | --- | --- | --- | --- | --- |
| **Cancer Type** | **Number** ^a^ | **Gene Name** | **Normal** ^b^ | **Cancer** ^b^ | **log2Fold Change** ^e^ | **Adjusted P value** ^d^ |
| **CRC** | 32 | ANGPTL5 | 8.17 | 0.33 | -4.64 | P<0.0001 |
|  |  | ANGPTL7 | 116.07 | 4.27 | -4.77 | P<0.0001 |
| **BRCA** | 113 | ANGPTL2 | 5381.78 | 1857.38 | -1.53 | P<0.0001 |
|  |  | ANGPTL4 | 1419.65 | 326.79 | -2.12 | P<0.0001 |
|  |  | ANGPTL5 | 40.76 | 2.09 | -4.28 | P<0.0001 |
|  |  | ANGPTL7 | 196.91 | 6.25 | -4.98 | P<0.0001 |
| **KIRC** | 72 | ANGPTL2 | 785.91 | 2200.69 | 1.49 | P<0.0001 |
|  |  | ANGPTL3 | 697.89 | 251.44 | -1.47 | P<0.0001 |
|  |  | ANGPTL4 | 570.73 | 18598.13 | 5.03 | P<0.0001 |
|  |  | ANGPTL6 | 13.86 | 4.12 | -1.75 | P<0.0001 |
|  |  | ANGPTL7 | 20.06 | 2.50 | -3.01 | P<0.0001 |
| **LUSC** | 51 | ANGPTL5 | 16.86 | 1.83 | -3.20 | P<0.0001 |
|  |  | ANGPTL7 | 62.72 | 2.72 | -4.53 | P<0.0001 |
| **THCA** | 59 | ANGPTL4 | 437.71 | 1142.75 | 1.38 | P<0.0001 |
|  |  | ANGPTL5 | 13.45 | 2.37 | -2.51 | P<0.0001 |
| **STAD** | 32 | ANGPTL3 | 21.55 | 2.63 | -3.03 | 0.04 |
|  |  | ANGPTL4 | 602.98 | 248.31 | -1.28 | 0.02 |
|  |  | ANGPTL7 | 134.24 | 16.31 | -3.04 | P<0.0001 |
| **LUAD** | 58 | ANGPTL4 | 454.62 | 1096.60 | 1.27 | 0.01 |
|  |  | ANGPTL5 | 16.80 | 2.58 | -2.70 | 0.0002 |
|  |  | ANGPTL7 | 68.37 | 3.50 | -4.29 | P<0.0001 |
| **CHOL** | 9 | ANGPTL3 | 19709.11 | 212.06 | -6.54 | P<0.0001 |
|  |  | ANGPTL4 | 5375.45 | 709.38 | -2.92 | P<0.0001 |
|  |  | ANGPTL6 | 820.64 | 10.29 | -6.32 | P<0.0001 |
| **KICH** | 25 | ANGPTL2 | 927.18 | 424.13 | -1.13 | 0.0002 |
|  |  | ANGPTL3 | 575.36 | 2.61 | -7.79 | P<0.0001 |
|  |  | ANGPTL6 | 28.91 | 1.53 | -4.24 | P<0.0001 |
| **BLCA** | 19 | ANGPTL2 | 3172.24 | 1069.43 | -1.57 | 0.006 |
|  |  | ANGPTL5 | 13.04 | 0.15 | -6.40 | 0.0003 |
|  |  | ANGPTL7 | 113.20 | 4.79 | -4.56 | P<0.0001 |
| **HNSC** | 43 | ANGPTL5 | 13.31 | 0.60 | -4.47 | P<0.0001 |
|  |  | ANGPTL7 | 98.49 | 10.33 | -3.25 | 0.02 |
| **KIRP** | 72 | ANGPTL3 | 862.78 | 26.57 | -5.02 | P<0.0001 |
| **LIHC** | 50 | ANGPTL3 | 17215.26 | 8426.23 | -1.03 | P<0.0001 |
|  |  | ANGPTL6 | 834.84 | 116.66 | -2.84 | P<0.0001 |

**Abbreviations:** CRC, Colorectal cancer; BRCA, Breast invasive carcinoma; KIRC, Kidney renal clear cell carcinoma; LUSC, Lung squamous cell carcinoma; THCA, Thyroid carcinoma; STAD, Stomach adenocarcinoma; LUAD, Lung adenocarcinoma; CHOL, Cholangiocarcinoma; KICH, Kidney chromophobe; BLCA, Bladder urothelial carcinoma; HNSC, Head and neck squamous cell carcinoma; KIRP, Kidney renal papillary cell carcinoma; LIHC, Liver hepatocellular carcinoma.

^a^ Number of paired normal and cancer tissues included in this study. We only considered the cancer type with six or more samples for differential expression analysis.

^b^ Mean expression level of normal or cancer tissue.

^c^ Fold change is calculated by mean expression level of Cancer/Normal.

^d^ P value was performed by DEGSeq package for R/Bioconductor and adjusted P value was conducted according to false discovery rate.
