# Supplementary material for: ANGPTL1 attenuates colorectal cancer metastasis by up-regulating microRNA-138
Source: J Exp Clin Cancer Res. 2017 Jun 12;36:78. doi: 10.1186/s13046-017-0548-7 (PMC5467265; doi:10.1186/s13046-017-0548-7)
Supplement: Supplementary file 2 — Basic characteristics of CRC patients in our center. (DOCX 35 kb) [file 13046_2017_548_MOESM2_ESM.docx]

|  |  | |
| --- | --- | --- |
| **Table S2. Basic characteristics of CRC patients in our center.** | | |
| Mean age(SD) | | 59.9±11.2 |
| Sex(%) |  | |
| Male | 27(57.4%) | |
| Female | 20(42.6%) | |
| UICC stage(%) | | |
| I | 12(25.5%) | |
| II | 11(23.4%) | |
| III | 11(23.4%) | |
| IV | 13(27.7%) | |
