# Supplementary figures and images for: ANGPTL1 attenuates colorectal cancer metastasis by up-regulating microRNA-138
Source: J Exp Clin Cancer Res. 2017 Jun 12;36:78. doi: 10.1186/s13046-017-0548-7 (PMC5467265; doi:10.1186/s13046-017-0548-7)

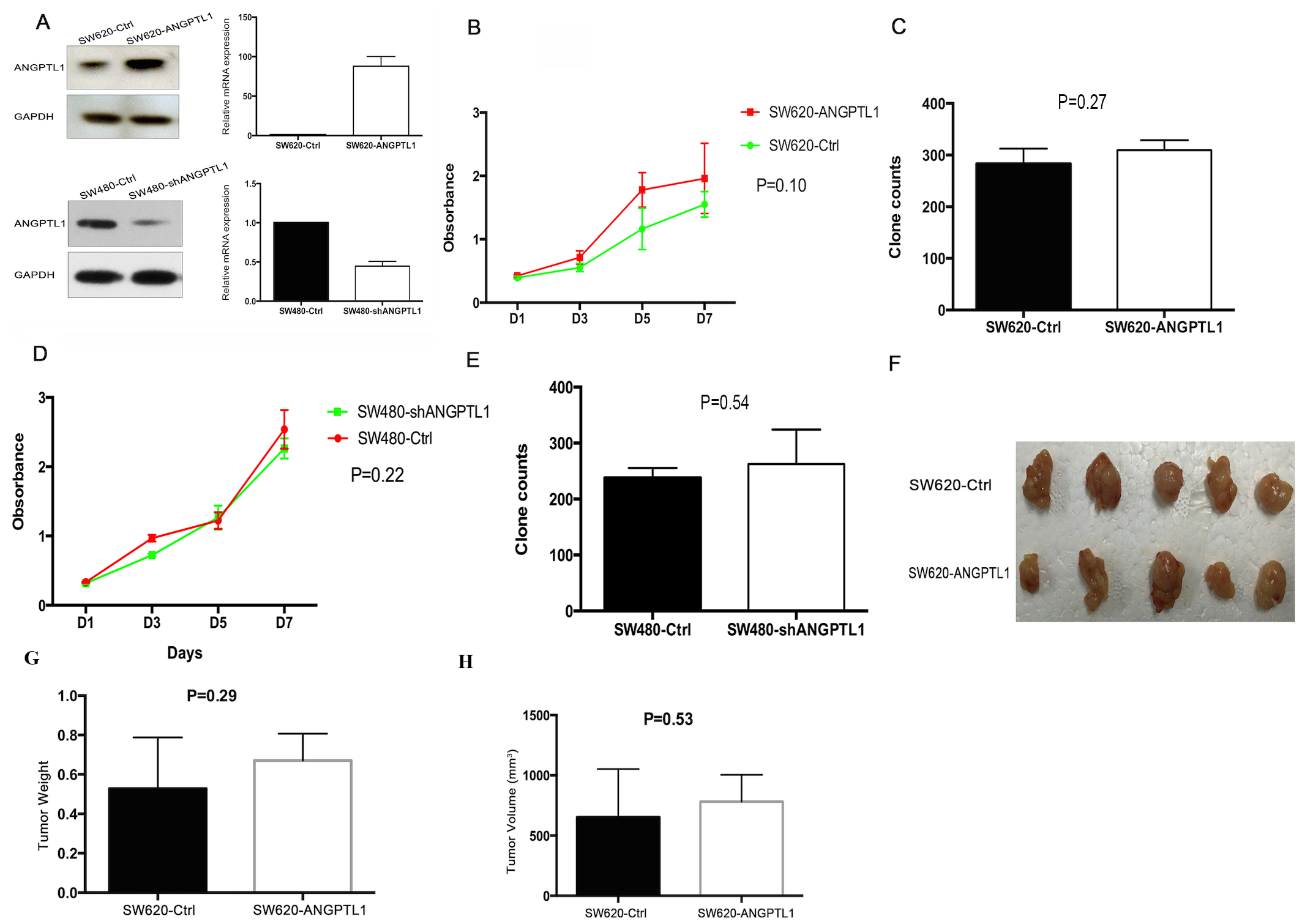

Supplement: Supplementary file 3 — ANGPTL1 does not affect the proliferative and colony-forming capacity of CRC cells. A. Generation of ANGPTL1-overexpressing/knockdown cell lines. SW620-ANGPTL1 cells exhibited an increase in ANGPTL1 at mRNA and protein levels, whereas ANGPTL1 expression was decreased in SW480-shANGPTL1 cells. B. Cell proliferation assay showed no significant difference between SW620-ANGPTL1 and SW620-Ctrl cells (P = 0.10). C. No significant difference was found by colony formation assay as well (P = 0.27). D. Cell proliferation assay showed no significant difference between SW480-shANGPTL1 and SW480-Ctrl cells (P = 0.22). E. Colony formation assay also revealed no significant difference between these two groups (P = 0.54). F. Subcutaneous tumor formed by SW620-ANGPTL1 and SW620-Ctrl cells. G-H: Tumor weight (P = 0.29) (G) and volume (P = 0.53) (H) were not significantly different between these two groups. (PNG 702 kb) [file 13046_2017_548_MOESM3_ESM.png]

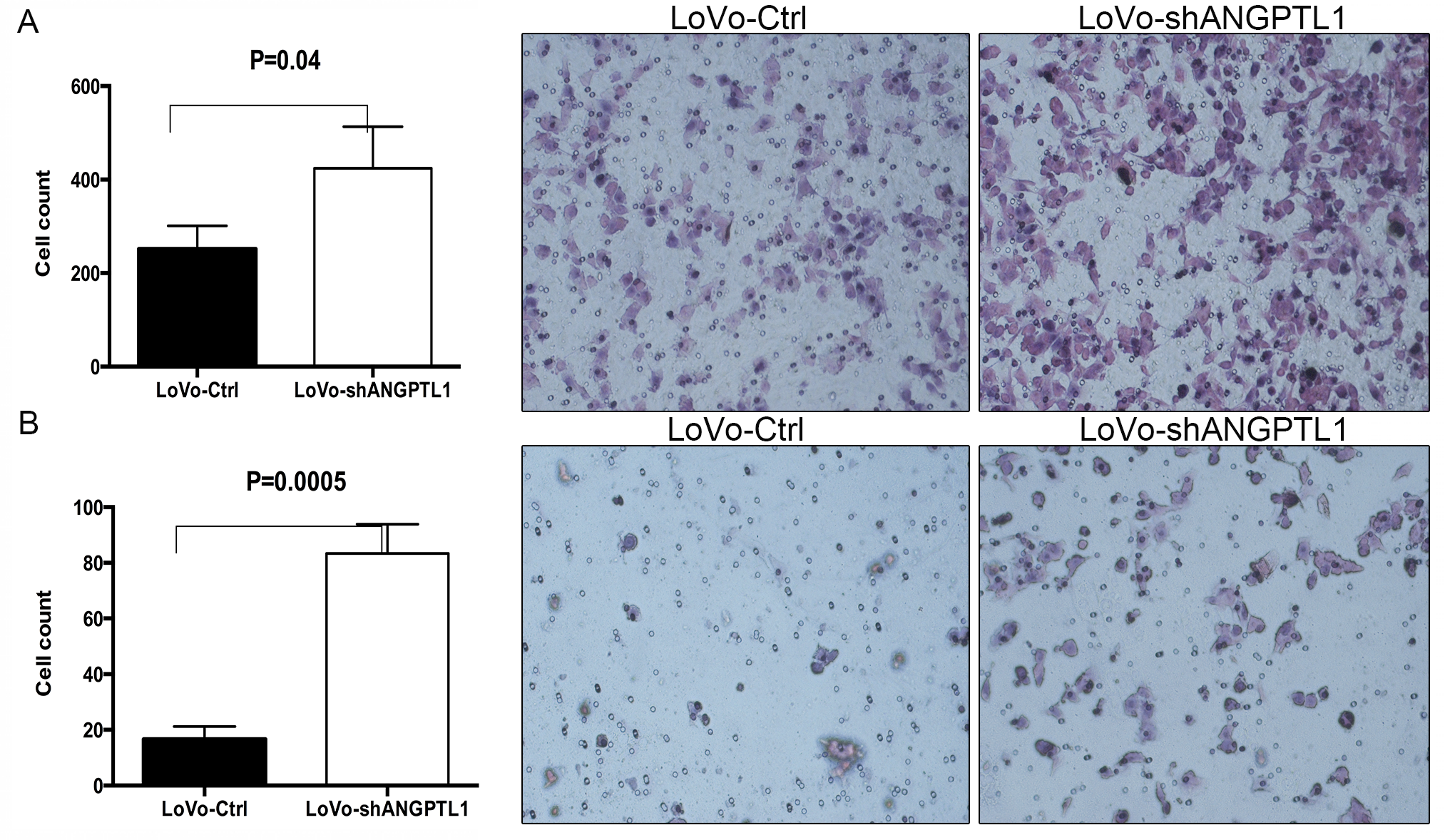

Supplement: Supplementary file 4 — ANGPTL1 knockdown promotes migration and invasion in LoVo cells. A. The number of invading cells was significantly higher in the LoVo-shANGPTL1 cells compared to that in the LoVo-Ctrl cells by transwell migration (P = 0.04) (A) and invasion assay (P = 0.0005) (B). (PNG 2705 kb) [file 13046_2017_548_MOESM4_ESM.png]
